# Fostering Maternal and Newborn Care in India the *Yashoda* Way: Does This Improve Maternal and Newborn Care Practices during Institutional Delivery?

**DOI:** 10.1371/journal.pone.0084145

**Published:** 2014-01-15

**Authors:** Beena Varghese, Reetabrata Roy, Somen Saha, Sidsel Roalkvam

**Affiliations:** 1 Public Health Foundation of India, New Delhi, India; 2 London School of Hygiene and Tropical Medicine, London, United Kingdom; 3 Indian Institute of Public Health Gandhinagar, Gujarat, India; 4 Centre for Development and Environment, University of Oslo, Oslo, Norway; Iran University of Medical Sciences, Iran (Republic Of Islamic)

## Abstract

**Background:**

The *Yashoda* program, named after a legendary foster-mother in Indian mythology, under the Norway-India Partnership Initiative was launched as a pilot program in 2008 to improve the quality of maternal and neonatal care at facilities in select districts of India. *Yashodas* were placed mainly at district hospitals, which are high delivery load facilities, to provide support and care to mothers and newborns during their stay at these facilities. This study presents the results from the evaluation of this intervention in two states in India.

**Methods:**

Data collection methods included in-depth interviews with healthcare providers and mothers and a survey of mothers who had recently delivered within a quasi-experimental design. Fifty IDIs were done and 1,652 mothers who had delivered in the past three months were surveyed during 2010 and 2011.

**Results:**

A significantly higher proportion of mothers at facilities with *Yashodas* (55 percent to 97 percent) received counseling on immunization, breastfeeding, family planning, danger signs, and nutrition compared to those in control districts (34 percent to 66 percent). Mothers in intervention facilities were four to five times more likely to receive postnatal checks than mothers in control facilities. Among mothers who underwent cesarean sections, initiation of breastfeeding within five hours was 50 percent higher in intervention facilities. Mothers and families also reported increased support, care and respect at intervention facilities.

**Conclusion:**

Yashoda as mothers' aide thus seems to be an effective intervention to improve quality of maternal and newborn care in India. Scaling up of this intervention is recommended in district hospitals and other facilities with high volume of deliveries.

## Introduction

Providing health care services, especially maternal and newborn care, is increasingly understood to be a dynamic system of entitlement and obligations among people, communities, providers, and governments. The paradox is that global community still concentrates on efforts to attain health-related Millennium Development Goals (MDGs) based on national strategies to reach high and equitable coverage of health services. The coverage of health services, though absolutely necessary, is not sufficient to attain the goals. The quality of treatment and care provided by health system can be complementary to the global efforts to reach and maintain coverage of health services. However, straight-forward indicators that can measure the quality of care are still to be identified 1].

The Janani Suraksha Yojana (JSY) launched by the Government of India in 2005 under the ambit of the National Rural Health Mission (NRHM) has resulted in unprecedented increase in institutional deliveries in India. The JSY beneficiaries increased from 700,000 in 2005–2006 to 9.23 million women in 2009–2010 2]. This dramatic increase in facility births, although a significant public health achievement, has now put tremendous pressure on the health institutions. The public health facilities lack infrastructure, manpower and other facilities to coordinate and ensure quality service delivery.

As a response, in 2006, the Norway-India Partnership Initiative (NIPI), a joint venture between the governments of Norway and India, was launched to provide catalytic and strategic support to NRHM in five focus states (more details available at NIPI.org.in). The specific aim of NIPI was to improve child health and related maternal health service delivery quality and access through facility and community-based interventions and through techno-managerial support at district and sub-district levels. NIPI introduced an innovative concept of a facility-based support worker or birth companion in facilities with high delivery volumes, named *Yashoda* (after a legendary foster mother of Indian mythology)—the focus of this paper.

A *Yashoda's* main role was to support the mother and the newborn child and assist the nurse in providing various non-clinical activities from the time the pregnant woman enters the facility till she leaves the hospital with the baby. The *Yashoda* thus is envisaged primarily as a mother's aide and birth companion. During this period, the *Yashoda* is to:

- Support the mother for immediate and exclusive breastfeeding;
- Orient the mother about basic newborn care and immunization;
- Assist the nurse in various postnatal care activities for making the newborn and the mother comfortable.
- Counsel the mother on family planning options, newborn care, nutrition, feeding practices, and hygiene.

The rationale for the *Yashoda* intervention is found in concepts such as baby-friendly hospital and mother-friendly health care complemented with continuum of care approach 3]. The evidence about the usefulness of birth companions who provide support to women during childbirth, range from psycho-social support to assistance with information and procedures 4,5]. Birth companions were traditionally community women or family members who comforted and supported a woman emotionally as she went through the stressful experience of childbirth.

Research since the 1970s has shown that the presence of a birth companion is extremely beneficial in easing the trauma of childbirth for the mother and in helping her cope with her experience 6]. Birth companionship was found to be positively associated with reduced length of labor and improved maternal-infant interaction 7]. A Cochrane review of 16 trials involving female birth companions found that women who had continuous intra-partum support were likely to have: slightly shorter labor, spontaneous vaginal birth, and less likely to have intra-partum analgesia 8]. Birth companions' presence is also likely to lead to fewer newborn complications 9].

In India, the Government of Tamil Nadu initiated a “birth companion” scheme in 2004 in all public hospitals in the state, under which women getting admitted to facilities could nominate a female family member to be their birth companion. A study there showed that the presence of a birth companion in the labor room may have reduced the likelihood of abuse by providers of women in labor 10].

This study is aimed to understand the space of a birth companion, *Yashoda*, in the limits of the maternities in select districts of Rajasthan and Odisha. The objective of this study thus was to assess the effectiveness of the *Yashoda* intervention in improving maternal and newborn care in Rajasthan and Odisha, two states of India. In Rajasthan, the *Yashodas* were placed in the district hospital (DH) as well as in some community health centers (CHCs) with high delivery load (300 to 1000 deliveries in a month); however, in Odisha this intervention was restricted to DH only with a delivery load of 500 per month.

## Methods

**Figure 1:**
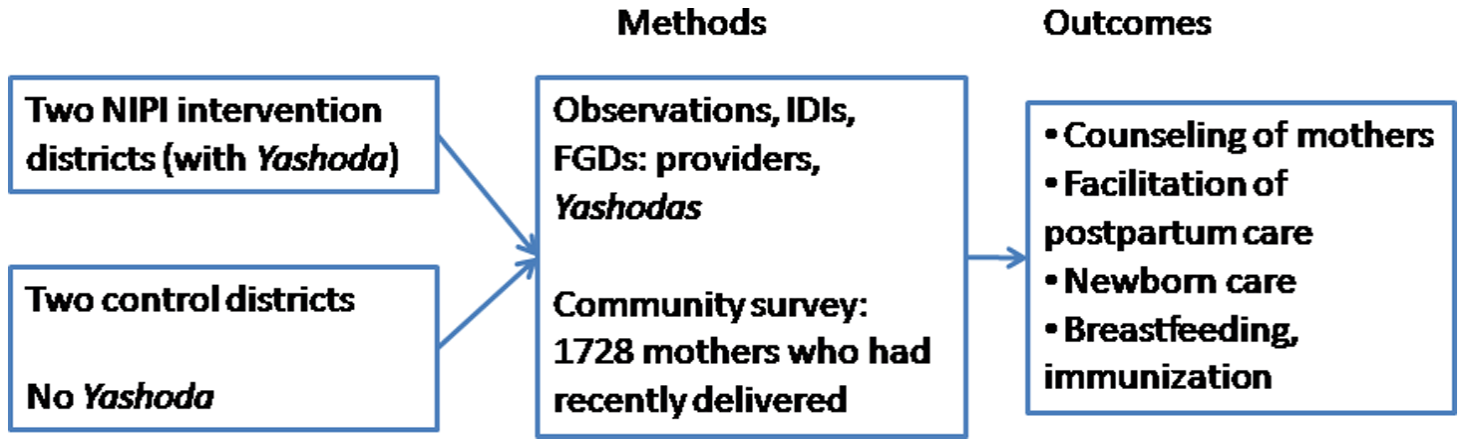
Study Framework: Quasi-experimental evaluation of the Yashoda program.

**Figure 2:**
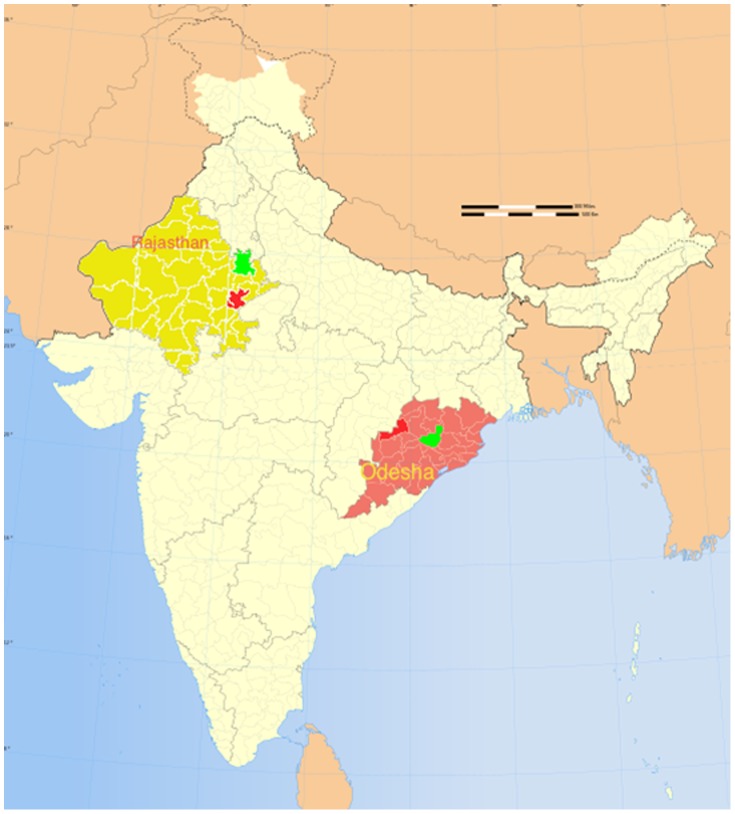
Map of India with study districts in Rajasthan and Odisha.

This independent evaluation study was conducted from January 2010 to September 2011 and used a multidisciplinary approach within a quasi-experimental design (intervention and control districts) to assess and evaluate two NIPI interventions – the *Yashoda* program and the home-based newborn care support provided by ASHAs (Figure 1). The current paper focuses on the *Yashoda* intervention. The intervention districts where the *Yashoda* program was fully functional were Alwar in Rajasthan and Anugul in Odisha; control districts with no *Yashodas* were Sawai Madhopur in Rajasthan and Bargarh in Odisha (Figure 2). The control districts were matched with NIPI intervention districts using DLHS III and census 2011 data 11,12] based on socioeconomic and epidemiological indicators (population density, literacy rates, rates of antenatal care and institutional delivery) (Table 1). During the study period (2010 to 2011), other than the routine programs of the National Rural Health Mission (NRHM), no other interventions were being implemented in the study facilities.

**Table 1:** Comparison of Intervention and Control districts.

Indicators	Rajasthan		Odisha		
	Intervention (Alwar)	Control (Sawai Madhopur)	Intervention (Anugul)	Control (Bargarh)	Source
**Population Characteristics**					
Population	2,990	1,117	1140.0	1346.0	Census 2001
Sex Ratio	887	889	941	976	Census 2001
Percent Rural population	85.5	81.0	86.1	92.3	Census 2001
Female Literacy Rate (7 years and above)	44.0	35.4	55.4	50.3	Census 2001
Current Use: Any Method (percent)	61.2	53.1	51.7	44.6	DLHS III
**Maternal Health**:					
Mothers registered in the first trimester when they were pregnant with last live birth/still birth (percent)	21.7	26.7	57.3	59.0	DLHS III
Mothers who had at least 3 Ante-Natal care visits	14.4	18.1	60.4	64.3	DLHS III
Institutional births (percent)	45.9	48.6	40.7	43.6	DLHS III
Delivery at home assisted by skilled personnel (percent)	10.6	8.5	11.5	14.2	DLHS III
Mothers who received post natal care within 48 hours of delivery of their last child (percent)	28.3	28.8	97.9	92.2	DLHS III
**Child Immunization**					
Children (12–23 months) fully immunized	25.1	26.4	62.0	70.4	DLHS III

Data collection methods included ethnographic studies, in-depth interviews (IDIs), focus group discussions (FGDs), and community survey. Community survey remained the main source of evaluation data. Ethnographic studies were conducted in two communities within the same district in Rajasthan where two fieldworkers lived, observed, and participated in community life. The two communities, though similar in economic status and in access to health care services, differed clearly in utilization of health care services.

The IDIs and FGDs were conducted by pre-trained facilitators and were based on a semi-structured guide covering the various issues to identify missing or weak links (bottlenecks) in the functioning of NIPI interventions. Local languages were used in all interviews and groups discussions, later translated and transcribed. The taped discussions were translated into English and transcribed into Microsoft Word by the facilitator and note takers' teams. Thematic analysis was coded and done manually by three investigators. The themes were initially analyzed in the form of role-ordered matrices, based on qualitative frameworks suggested by Miles and Huberman 13]. Saturation was achieved on the main themes. Findings from interviews help guide the survey questionnaire.

The community survey was conducted between March to May 2011 in all study districts. For the community survey, the study participants were defined as ‘mothers who delivered at district hospitals in the last three months preceding the survey.’ To calculate the required sample size for the community survey, the proportion of mothers who initiated breastfeeding within one hour of delivery (IBF1) was assumed to be 60 percent. To demonstrate at least a 25 percent difference between the intervention and control groups (with 80 percent power and α = 0.05), the minimum sample was estimated to be 216 mothers per arm. A design effect of two was assumed, increasing the sample size to 432 per arm.

The survey conducted in two states included a total sample of 1,728 mothers across the intervention and control districts. A detailed questionnaire was developed for the community survey, divided into thematic sections. Mothers were specifically asked regarding receipt of practices or services that were specific to *Yashoda's* tasks (listed below).

Based on the job profile of the *Yashodas*, the primary indicators for the program included:

1. Counseling of mothers on exclusive breast feeding, family planning, nutrition, danger signs, cleanliness;
2. Facilitation of immediate postpartum care for mother and new born;
3. Initiation of breastfeeding within one to five hours after delivery;
4. Weighing of the baby.
5. Immunization with birth dose of Polio and BCG.

Explanatory variables included indicators of demographic and socio-economic status and maternal and child outcomes. Details of pregnancy history and birth experience were also collected, including antenatal, intranatal and postnatal care, quality of care (cleanliness, availability of toilet and drinking water), trust and emotional support, cost of care and awareness and receipt of JSY scheme.

The survey data was analyzed using SPSS version 18. Descriptive analyses and bivariate analyses were followed by binary logistic regression analyses to estimate the effect (through adjusted odds ratios) of *Yashodas* on maternal and newborn indicators. The equation used was:
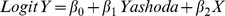



Where *Y* is the outcome of interest, *Yashoda* takes value 1 if respondent was from intervention area and exposed to an *Yashoda*, X is a vector for control variables (age, education, income and type of deliveries, and number of ANC visits) while, (β_1_ and β_2_) are maximum likelihood estimates of the logistic regression coefficients. Adjusted odds ratios were reported as increased or decreased likelihood of occurrence of an event. Eighteen specifications have been used for each state: Six counseling variables (exclusive breast feeding, family planning, immunization, nutrition, identification of danger signs, hygiene); Six postpartum care variables (blood pressure check, temperature check, perineum check, episiotomy check, check for injection, and saline check); and Six newborn care practice variables (mothers who took measures to keep newborn warm, were provided with food and water at PNC ward, initiated breastfeeding within one hour, initiated breastfeeding within 1–5 hours, did not gave supplementary feed to newborn, and received first immunization doses).Thus, for example, for the indicator, exclusive breast feeding (dependent variable): probability or odds of exclusive breast feeding for mothers exposed to *Yashodas* compared to those without *Yashoda* exposure is calculated adjusted for age, education, income, type of delivery, and number of ANC visits.

### Ethics Statement

For in-depth interviews (IDI), most health care providers gave written consent; mothers in the community gave verbal informed consent after the purpose and proceedings of the study were explained to them, for some participants who did not want their conversations recorded, researchers took notes. Written consent was obtained for almost all IDIs with providers. Some doctors were not comfortable with providing written consent so we took verbal consent and clearly told them (as well as all others) that they are free to stop the interview or not answer any question if they did not wish to. It was clarified that in the final report or in publications, no name will be identified.

For mothers, an information sheet about the study was read, and told that they are free to answer all or part of the questionnaire and at any time they felt uncomfortable, they can stop the interview. Since most mothers were illiterate or had minimal education it was deemed not useful to collect written consent. ASHAs and Aganwadi workers (community level workers) were informed regarding the survey and they were asked to inform mothers about the same and were provided with a study information sheet.

For IDIs with providers, signed written consent forms were collected, for the two doctors who did not provide written consent; the interviewer noted the same in the recording. For survey of mothers, each data collector noted in the information sheet that verbal consent was taken.

The Institutional Ethics Committee at the Public Health Foundation of India was provided with a copy of information sheet and informed consent forms. The ethics application form clearly stated that for the survey of mothers in the community, verbal consent would be obtained after informing them about the study and their ability to stop the process at any time. The committee approved the research protocol, including the consent process.

## Results

### Characteristics of the Sample

The community survey provided valid responses from 1,652 women (out of the 1,750 mothers interviewed), 810 in intervention and 842 in control districts, with a response rate of 94 percent (Table 2). The median age of respondents ranged from 22 to 24 years, education levels were similar across intervention and control groups within a state, however quite different between Rajasthan and Odisha. Within Rajasthan, Alwar with its more urban population reported slightly higher levels of educated women than the control area (25% of women in Alwar reported having more than eighth grade education compared to 14% in Sawai Madhopur. A larger proportion of mothers in Rajasthan reported living in *pucca* houses than those in Odisha. Women in intervention district, Alwar (Alwar is more urban than Sawai Madhopur) reported higher levels of education than in control district of Sawai Madhopur. Nurses conducted 70 to 84 percent of the deliveries, with no significant differences between intervention and control groups. Family members influenced place of delivery for most, and the biggest reason for choosing a place of delivery was the perception of ‘good facility’ as reported by 66 percent respondents; cost was the second important factor. More mothers in Odisha than in Rajasthan (83 percent to 86 percent vs. 57 percent to 69 percent) received more than three ANC visits (Table 2).

**Table 2:** Key indicators of socio-economic and reproductive health characteristics of respondents.

	Rajasthan		Odisha	
Characteristics	Intervention Alwar	Control Sawai Madhopur	Intervention Anugul	Control Bargarh
Number of respondents	451	489	359	353
Median age (years)	22	24	23	24
Median monthly household income (INR)	7000	6500	4500	4000
Level of education (percent):				
No formal education	29	33	12	16
1st–8th grade	47	54	47	44
9th – 12th grade	17	11	38	33
Higher than 12th grade	8	3	3	7
Type of House				
Pucca	80	63	46	35
Semi-pucca	15	25	19	18
Kuccha	5	12	35	48
Birth order				
1^st^	32	24	45	41
2^nd^	29	26	33	30
3^rd^	18	20	13	18
4th and above	21	30	10	11
Assistance during delivery				
Doctors	23	31	57	65
Nurses	70	79	84	81
ASHA/Dais	15	10	15	4
Three or more ANC visits	69	57	83	86
Place of ANC				
DH/SDH/CHC	38	29	41	66
SC/AWC	41	50	20	22
Private facility	10	24	65	37
Home	20	12	1	1

### Yashoda Characteristics

As envisaged by NIPI, *Yashodas* provided care and support to mothers and newborns in select district hospitals in Rajasthan and Odisha. They were provided a pink apron or sari to ensure a separate identity at the facilities. One Yashoda attended five to six mothers and their newborns during an eight-hour shift. The median age of a *Yashoda* was 33 years in Odisha and 35 years in Rajasthan. The adopted remuneration model was different in the two states. In Rajasthan, payment to *Yashodas* was based on the number of deliveries in the hospital (an incentive of 100 Indian Rupees ($2) per conducted institutional delivery) with a reported median of Rs. 4,000 ($80) per month. In Odisha, a fixed amount of Rs. 3,000 ($60) per month was paid to them. Most of the *Yashodas*, however, preferred a combined model for remuneration—a fixed amount plus incentives linked to performance rather than to number of deliveries.

Most (75 percent) of *Yashodas* received two to three days training on counseling and on the nature of her supportive role at the facility for mother and newborn. The Yashodas found the training sessions helpful in defining their role clarity and dispensing their duties. Most of the Yashodas recommended continued refresher training to upgrade their skills.

*“Through this training we got knowledge about family planning, immunization, breast feeding, diet of the mother, how to receive a mother and child after delivery, how to maintain hygiene within the hospital, what are the problems that a mother faces after delivery etc.” (IDI, Yashoda, Alwar and Anugul).*

In both the states, the supervisors (generally a retired auxiliary nurse midwife or nurse) were appointed either along with or before the Yashodas were appointed and provided support to them. This was very much appreciated by the Yashodas.

*“She supervises our work personally. She interacts with mothers and asks them what information they have received from the Yashoda. She suggests us in which way we can do our work better.” (IDI, Yashoda, Alwar)*

### Counseling and Support in Facility

Yashodas spend most of their time, almost forty percent, in the labor room and in the postnatal ward. Although, one of their responsibilities was to be available as a mother comes to the facility for registrations, almost no interactions with mothers were reported during the registration process. Eighty one percent of mothers in Alwar and 93 percent in Anugul reported interacting with *Yashodas* only in the PNC ward. In the PNC ward, Yashodas reported spending most of their time on counseling mothers on breastfeeding, nutrition, family planning, hygiene, identification of danger signs, and on immunization. This was corroborated by mothers too–mothers in the intervention areas were two to nine times more likely to receive counseling on these topics than those in control areas (Tables 3 and 4). The proportion of mothers who reported receiving counseling messages, however, varied across the topics. For example, exclusive breast-feeding was the most discussed among the six topics, with 95 percent of the respondents in Alwar reporting having received information on it (9.07 [95 percent CI 5.71–14.41]); however, only 55 percent of mothers reported receiving any information on danger signs at the facility. This variation was much smaller in Anugul, the intervention district of Odisha, where the proportions of counseling by topic ranged from 83 percent to 97 percent (Tables 3 and 4).

**Table 3:** Impact of Yashoda on counseling, postpartum checkup and newborn care, Rajasthan.

	District Hospital		Adjusted Odds Ratio^#^(95 percent CI)
	Intervention (n = 207)	Control (n = 204)	
**COUNSELING**			
Exclusive breast feeding	95 **	34	9.07 (5.71–14.41)
Family planning	68 *	56	2.48 (1.78–3.46)
Immunization	92 **	48	5.48 (3.63–8.26)
Nutrition	81 **	66	1.97 (1.38–2.82)
Identification of danger signs	55 **	46	2.83 (2.02–3.97)
Cleanliness/hygiene	76 **	36	3.19 (2.27–4.47)
**POST NATAL CARE**			
Blood pressure check	48 **	14	4.13 (2.79–6.13)
Temperature check	31**	6	5.95 (3.54–10.00)
Perineum check	33 **	15	1.99 (1.32–2.99)
Episiotomy check	34*	19	1.62 (1.07–2.44)
Check for injection	42	33	1.29 (0.96–1.72)
Saline check	23 **	9	2.74 (1.71–4.37)
**NEWBORN CARE**			
Mothers who took measures to keep the newborn warm	91	99	0.48 (0.27–0.86)
Mothers who were provided food and water at the PNC ward	81	97	0.31 (0.18–0.56)
Initiation of breast feeding within 1 hour	41	39	1.08 (0.78–1.49)
Initiation of breast feeding from 1–5 hours	49	44	1.13 (0.82–1.56)
Mothers who did not gave supplementary feed to newborn	28*	17	1.67(1.13–2.47)
Newborns who received first immunization – BCG and OPV(0)	98	93	1.32 (0.78–2.23)

# Adjusted for age, education, income, type of delivery, and number of ANC visits.

p = <.05, **p = <.001.

**Table 4:** Impact of Yashoda on counseling, postpartum checkup and newborn care, Odisha.

	Intervention (n = 253)	Control (n = 251)	Adjusted Odds Ratio^#^(95 percent CI)
**COUNSELING**			
Exclusive breast feeding	97	94	1.89 (0.77–4.66)
Family planning	84 **	66	3.12 (2.06–4.73)
Immunization	96 *	91	2.34 (1.11–4.94)
Nutrition	83	78	1.29 (0.84–1.97)
Identification of danger signs	85 **	49	4.74 (3.14–7.17)
Cleanliness/hygiene	95 **	78	3.91 (2.09–7.31)
**POST NATAL CARE**			
Blood pressure check	29 *	20	1.50 (1.03–2.19)
Temperature check	23 *	15	1.39 (0.93–2.08)
Episiotomy stitches	29 **	12	2.49 (1.64–3.78)
Perineum check	27	20	1.33 (0.91–1.95)
Injection	45	45	1.28 (0.92–1.78)
Saline	38 *	27	1.50 (1.05–2.12)
**NEWBORN CARE**			
Mothers who took measures to keep the newborn warm	97	94	1.65 (0.70–3.90)
Mothers who were provided food and water at the PNC ward	95 **	81	5.70 (2.99–10.82)
Initiation of breast feeding within 1 hour	78	73	1.09 (0.73–1.63)
Initiation of breast feeding from 1–5 hours	17	14	1.60 (1.00–2.56)
Mothers who did not give supplementary feed to newborn	26	21	1.31 (0.92–1.87)
Newborns who received first immunization – BCG and OPV(0)	93**	73	5.26 (3.08–8.99)

# Adjusted for age, education, income, type of delivery, and number of ANC visits.

p = <.05, **p = <.001.

**Figure 3:**
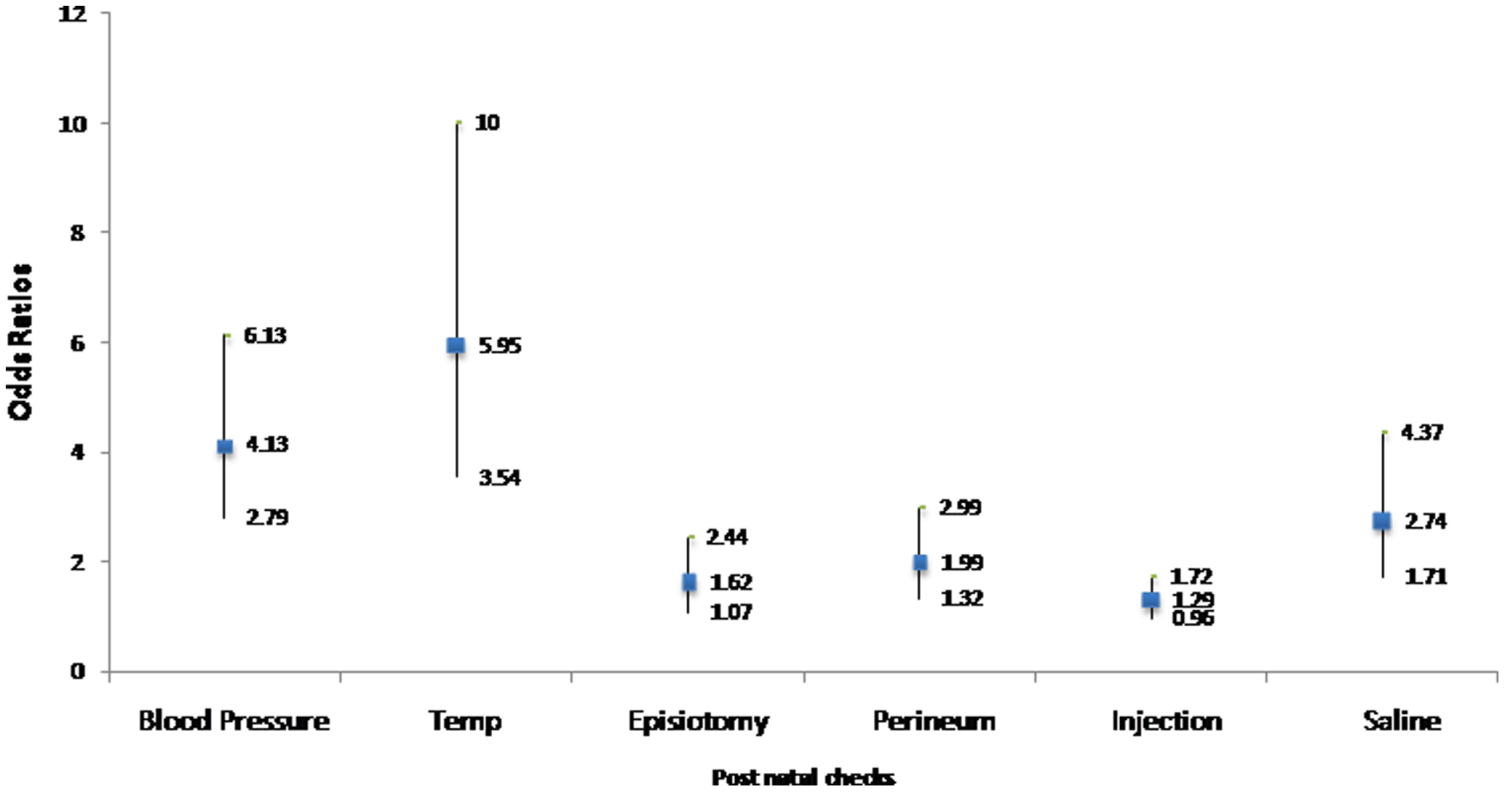
Yashoda Effect on postnatal care: Odds ratios with 95 percent Cl, Rajasthan.

Yashodas facilitated some of the essential postnatal care such as checking the temperature, blood pressure and perineum for mothers at the facilities: 48 percent of the mothers in Alwar DH reported checking of blood pressure compared to 14 percent in Sawai Madhopur (29 percent in Anugul, 20 percent in Bargarh). Similarly, mothers at the Alwar DH were 5.95 times (95percent CI 3.54 to 10) more likely to have their temperature checked than those in Sawai Madhopur without any *Yashodas*; mothers in Anugul were 2.49 times (95 percent CI 1.64 to 3.78) more likely to have their episiotomy stitches checked compared to mothers in Bargarh (Figure 3).

### Neonatal Care Practices

Some of the neonatal care practice indicators (keeping the newborn baby warm, providing food and water at the PNC ward, initiating breast feeding within one hour and immunization) did not always show significant differences between the intervention and control districts, especially in Rajasthan (Tables 3 and 4). In Odisha, some significant differences were observed in between NIPI intervention and non-NIPI areas, for example, neonates in Anugul were 5.26 times (95 percent CI 3.08to 8.99) more likely to receive birth dose BCG and OPV compared to those in the control district (Table 4).

The impact of Yashodas were most apparent for mothers with C-section delivery; 76 percent of respondents who had C-section delivery in the intervention districts reported that they initiated breast feeding within five hours compared to 44 percent (P<0.001) in the control district (Table 5).

**Table 5:** Key postnatal indicators for mothers who had a Cesarean-section delivery.

	Intervention (n = 46)	Control (n = 46)
Initiated breast feeding within 5 hours	76**	44
Mothers whose C-section scar was checked	96*	83
Mothers whose dressing was changed (for C-Section)	94*	72

p = <.05, **p = <.001.

This was mainly attributed to the support provided by Yashodas to position the baby in a less-painful manner and aiding early breastfeeding.

*“Yashoda told her the right way of breast feeding and about the family planning methods also. Yashoda continuously monitors for problems and checks if mother has any discomfort” (IDI, Caregiver, Anugul).*

Similarly, more than 95 percent of the respondents who had C-section delivery reported that their C-section scar was checked and that their dressings were changed compared to 83 percent and 72 percent in the control districts for the same indicators.

Women reported improved or better overall experience at facilities with yashodas compared to those without Yashodas. Mothers and families felt that the presence of Yashodas was beneficial to them in several ways. Mothers reported being more comfortable within the hospital environment, in the presence of *Yashodas*. It was pointed out that “….*even people from high socio-economic status don't want to stay in (an exclusive) cabin because there are no Yashoda services in the cabin.”*

*Yashodas* were eager and quick to help mother's breastfeed the newborn babies. They emphasized the importance of exclusively breastfeeding the baby to everyone including mothers-in-law and relatives. Existing nursing staff at the facilities also appreciated this new cadre of support workers.

*“..After the coming of Yashodas at the hospital, we have got much help from them, because, now we do not need to worry about mothers as Yashodas take care of the mothers…” (IDI, Staff nurse, Alwar DH).*

Sometimes *Yashodas* also acted as change agents.

## Discussion

This study found that *Yashodas* provided support and care to mothers and newborn babies; mothers and families felt that the presence of *Yashodas* increased the comfort level at facilities. A significantly higher proportion of mothers who delivered at facilities where *Yashodas* were present reported having received counseling on a variety of maternal and newborn care issues when compared to respondents who delivered at facilities where *Yashodas* were not present. More importantly, for mothers who had a cesarean section, presence of *Yashodas* significantly improved their ability to initiate breastfeeding within five hours of delivery.

*Yashodas* also enabled a significantly higher proportion of mothers to receive postnatal checks at the facility. However, this proportion that received basic postnatal checks at the intervention facilities is still quite low (ranging from 20 percent to 40 percent). This reflects the poor quality of PNC care currently available at these facilities. Improving postnatal care for mothers and newborn babies in all facilities would have a significant impact on maternal and neonatal mortality and morbidity. Although, the presence of *Yashodas* has improved the level of care, there is an immense scope for improvement in immediate postnatal care inside maternities, which should be universally received by mothers at all facilities.

Some of the neonatal care practice indicators (keeping the newborn warm, provision of food and water at the PNC ward, initiation of breast feeding within one hour and immunization) did not always show significant differences between the intervention and control districts and were reported by almost 80percent of mothers. This perhaps depicts the impact of efforts under NRHM to improve these neonatal care indicators across all facilities in India. NRHM programs have focused extensively on newborn care practices, mainly on initiation of breastfeeding and keeping the newborn warm.

It is important to note that the level of respondents' exposure to health personnel including the *Yashoda* is dependent on the length of stay at the facility. The community survey showed that 82 percent of mothers who had a normal delivery in Alwar and 24 percent of those in Anugul, stayed at the health facility for at least 48 hours after delivery. The length of stay at the facility therefore impacts the level of *Yashoda* exposure and the associated benefits. The length of stay however, is influenced by a variety of factors ranging from type of delivery, health system issues and influence of family members.

In both intervention and control districts 70 percent of mothers expressed confidence in the health facility by opining that they would go back to institutional delivery for their next pregnancy. The positive experience at the facility was an important reason for mothers in Rajasthan, especially in intervention area (73percent in Alwar versus 32 percent in Sawai Madhopur); whereas in Odisha, the incentive received through JSY was an important reason for women to return to facilities for their next delivery.

The strength of this study is its multi-method approaches with ethnography, qualitative (IDIs, FGDs) and quantitative (survey) methods. This study also has a few limitations: one of the main limitations was lack of baseline information on the selected indicators; however, this was partially addressed through the selection of control districts that most matched the intervention district. Another issue was the limited analysis on mothers who had cesarean section due to the small sample size. Contrary to our expectation, the number of mothers with C-sections was not very high at district hospitals. The study was designed to measure impact of *Yashodas* on maternal and neonatal care practices. Thus, the interpretation of the findings from this study thus should be limited to the impact on these indicators and not on neonatal mortality outcomes. A study modeling the potential impact of *Yashodas* on neonatal mortality is described elsewhere 14].

*Yashodas* thus appear to be an important cadre of workers who provide significant support to mothers and newborns in institutions—through improved counseling and facilitation of important postnatal care. Their support has shown to result in significantly higher levels of information among mothers (through counseling) and in improved immediate postpartum care. However, it is important to suitably highlight *Yashoda's* role as a mother's aide, possibly illustrating the importance of emotional support required for a satisfactory delivery experience and her crucial role in ensuring the same. This would help the *Yashoda* and the staff to understand and appreciate their role and create a unique identity for *Yashodas* in the facility.

This study suggests that *Yashodas* when placed at high delivery load facilities like a district hospital, provides support to mothers and new born; provides counseling to mothers on maternal and newborn care practices; and also facilitates immediate postpartum care to mother and newborn. Thus, the *Yashoda* intervention appears to be an effective intervention in improving the quality of maternal and newborn care during institutional delivery and could potentially have an impact on neonatal and maternal mortality. Thus, a scale up of this intervention especially in high delivery load facilities across India is highly recommended.

## References

[1] Graham WJ, Varghese B (2012) Quality, quality, quality: gaps in the continuum of care. The Lancet 379: e5–e6. 10.1016/S0140-6736(10)62267-2 21474173

[2] Dongre AA (2010) Effect of Monetary Incentives on Institutional Deliveries: Evidence from the Janani Suraksha Yojna in India.

[3] Unicef (1991) The Baby-Friendly Hospital Initiative. Unicef and World Health Organization.

[4] Koumouitzes-Douvia J, Carr CA (2006) Women's perceptions of their doula support. The Journal of perinatal education 15: 34. 10.1624/105812406X151402 PMC1804309 17768433

[5] Campero L, García C, Díaz C, Ortiz O, Reynoso SA, et al (1998) “Alone, I wouldn't have known what to do”: A qualitative study on social support during labor and delivery in Mexico. Social Science & Medicine 47: 395–403. 9681909 10.1016/s0277-9536(98)00077-x

[6] Government of Tamil Nadu (2006) Note on Birth Companionship Programme

[7] Sosa R, Kennell J, Klaus M, Robertson S, Urrutia J (1980) The effect of a supportive companion on perinatal problems, length of labor, and mother-infant interaction. New England Journal of Medicine 303: 597–600. 7402234 10.1056/NEJM198009113031101

[8] Hodnett ED, Gates S, Hofmeyr GJ, Sakala C (2007) Continuous support for women during childbirth. Cochrane Database Syst Rev 3. 10.1002/14651858.CD003766.pub2 17636733

[9] Leslie M, Storton S (2007) The Coalition for Improving Maternity Services: Evidence basis for the ten steps of mother-friendly care. Step 1: Offers all birthing mothers unrestricted access to birth companions, labor support, professional midwifery care. Journal of Perinatal Education 16: 10S–19S. 18523678 10.1624/105812407X173137 PMC2409134

[10] Subha Sri B (2009) Translating medical evidence into practice. In: Council P, editor. Working paper. Delhi.

[11] International Institute for Population Sciences (IIPS) (2010) District Level Household and Facility Survey (DLHS-3), 2007-08. Mumbai.

[12] Chandramauli C (2011) Census of India 2011: provisional population totals paper 1 of 2011 India Series 1, Chapter 6. New Delhi, India: Office of the Registrar General & Census Commissioner.

[13] Miles MB, Huberman AM (1994) Qualitative data analysis: An expanded sourcebook: Sage.

[14] Saha S, Varghese B (2013) Cost-effectiveness of Yashoda, a facility based mother and newborn support intervention in India. Submitted to Bulletin of World Health Organization.

